# Recent UK type 2 diabetes treatment guidance represents a near whole population indication for SGLT2-inhibitor therapy

**DOI:** 10.1186/s12933-023-02032-x

**Published:** 2023-11-02

**Authors:** Katherine G. Young, Rhian Hopkins, Beverley M. Shields, Nicholas J. Thomas, Andrew P. McGovern, John M. Dennis, the MASTERMIND consortium

**Affiliations:** Department of Clinical and Biomedical Sciences, RILD Building, Royal Devon & Exeter Hospital, University of Exeter Medical School, Barrack Road, Exeter, EX2 5DW UK

**Keywords:** Diabetes mellitus type 2, SGLT2-inhibitors, Cardiovascular disease

## Abstract

Recent type 2 diabetes guidance from the UK’s National Institute for Health and Care Excellence (NICE) proposes offering SGLT2-inhibitor therapy to people with established atherosclerotic cardiovascular disease (ASCVD) or heart failure, and considering SGLT2-inhibitor therapy for those at high-risk of cardiovascular disease defined as a 10-year cardiovascular risk of > 10% using the QRISK2 algorithm. We used a contemporary population-representative UK cohort of people with type 2 diabetes to assess the implications of this guidance. 93.1% of people currently on anti-hyperglycaemic treatment are now recommended or considered for SGLT2-inhibitor therapy under the new guidance, with the majority (59.6%) eligible on the basis of QRISK2 rather than established ASCVD or heart failure (33.6%). Applying these results to the approximately 2.20 million people in England currently on anti-hyperglycaemic medication suggests 1.75 million people will now be considered for additional SGLT2-inhibitor therapy, taking the total cost of SGLT2-inhibitor therapy in England to over £1 billion per year. Given that older people, those of non-white ethnic groups, and those at lower cardiovascular disease risk were underrepresented in the clinical trials which were used to inform this guidance, careful evaluation of the impact and safety of increased SGLT2-inhibitor prescribing across different populations is urgently required. Evidence of benefit should be weighed against the major cost implications for the UK National Health Service.

Recent type 2 diabetes guidance from the UK’s National Institute for Health and Care Excellence (NICE) proposes offering SGLT2-inhibitor therapy to people with established atherosclerotic cardiovascular disease (ASCVD) or heart failure, and considering SGLT2-inhibitor therapy for those at high-risk of cardiovascular disease defined as a 10-year cardiovascular risk of > 10% using the QRISK2 algorithm [1]. The guidance applies irrespective of glycaemic control and both for people initiating first-line anti-hyperglycaemic treatment and those established on therapy. By including those without established ASCVD, this guidance goes beyond the evidence from clinical trials of the cardiovascular benefit of SGLT2-inhibitors in which a majority of participants (57–85%) had established cardiovascular disease [2]. The implications of this proposal on national prescribing are not reported.

We used a contemporary population-representative UK cohort of people with type 2 diabetes (n = 568,524) actively registered with a GP practice in February 2020 (using Clinical Practice Research Datalink) to assess the implications of this guidance. 93.1% of people currently on anti-hyperglycaemic treatment are now recommended or considered for SGLT2-inhibitor therapy under the new guidance, with the majority (59.6%) eligible on the basis of QRISK2 rather than established ASCVD or heart failure (33.6%) (Fig. 1). Prescribing of SGLT2-inhibitors at the time of evaluation was limited to 14.9% of people on anti-hyperglycaemic treatment, of whom a minority had established ASCVD or heart failure (26.6%).

**Fig. 1:**
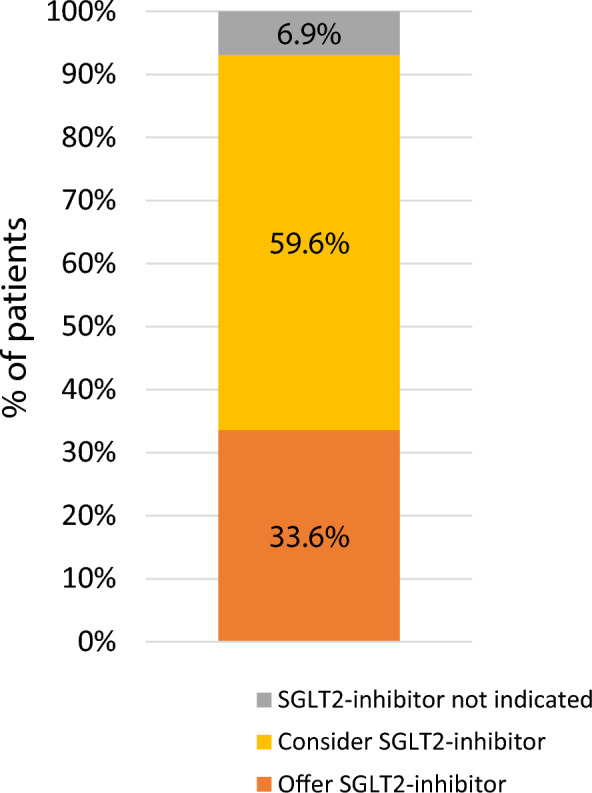
SGLT2-inhibitor recommendations for type 2 diabetes currently treated with anti-hyperglycaemic medication in UK primary care. Estimates are derived from a UK cohort of people with type 2 diabetes (Clinical Practice Research Datalink, n = 568,524 actively registered with a GP practice in February 2020). People with chronic kidney disease stage 4–5 were excluded (n = 18,594) as this group represents a specific population with different criteria for SGLT2-inhibitor initiation. Remaining individuals were classified as either currently receiving anti-hyperglycaemic treatment (73.0%, n = 415,267) or currently untreated (27.0%, n = 153,257). NICE criteria was then used to classify individuals by cardiovascular disease status as (1) having established atherosclerotic cardiovascular disease or heart failure [Offer SGLT2-inhibitor]; (2) at high-risk of cardiovascular disease as defined by a 10-year risk of cardiovascular disease > 10% applying the QRISK2 algorithm [Consider SGLT2-inhibitor]; (3) not at high-risk of cardiovascular disease (QRISK2 < 10%) [SGLT2-inhibitor not indicated]. Amongst the 27.0% currently untreated individuals, the majority of whom will eventually require treatment, 93.3% are recommended or considered for SGLT2-inhibitor therapy, with the majority (57.6%) eligible on the basis of QRISK2 rather than established ASCVD (35.7%) [data not shown]

Applying these results to the approximately 2.20 million people in England currently on anti-hyperglycaemic medication suggests 1.75 million people not currently receiving SGLT2-inhibitor therapy will now be considered for additional SGLT2-inhibitor therapy under the new guidance. Full implementation (prescribing of SGLT2-inhibitor therapy to all those recommended or considered for them) could increase the total cost of SGLT2-inhibitor prescribing from £147 million [3] to over £1 billion per year. Lower rates of prescribing among those considered for SGLT2-inhibitor therapy still represent substantial cost increases: approximately £700 million for 80% uptake, and £580 million for 60% uptake. For context, total annual spending on cholesterol-lowering drugs in the whole population with and without diabetes is around £215 million [4]. The cost of SGLT2-inhibitors is likely to reduce substantially when generic forms become available, as has been the case for statins [5].

The updated NICE guidance represents a potential near population-level intervention of SGLT2-inhibitor initiation for people with type 2 diabetes. Recommendations are based on extrapolation of trial evidence to people at lower cardiovascular risk. Given that older people and non-white ethnic groups are under-represented in trials, careful evaluation of changes in SGLT2-inhibitor prescribing across different populations, and the impact and safety of these changes [6], is urgently required. A full economic analysis is needed to assess the benefit (reduction in ASCVD/heart failure events) against the major cost implications for the UK National Health Service (NHS). If guidelines are adhered to, increases in SGLT2-inhibitor prescribing could near double the £1 billion annual spend on diabetes drug prescribing [7].

## Data Availability

Requests to access data provided by Clinical Practice Research Datalink (CPRD) should be directed to CPRD.

## Abbreviations

ASCVD: Atherosclerotic cardiovascular disease
GP: General practice
NICE: National Institute for Health and Care Excellence
NHS: National Health Service
SGLT2-inhibitor: Sodium-glucose transport protein 2 inhibitor
